# Capturing Patients' Perspectives on Medication Safety: The Development of a Patient-Centered Medication Safety Framework

**DOI:** 10.1097/PTS.0000000000000583

**Published:** 2019-03-15

**Authors:** Sally J. Giles, Penny J. Lewis, Denham L. Phipps, Faith Mann, Anthony J. Avery, Darren M. Ashcroft

**Affiliations:** From the ∗ NIHR Greater Manchester Patient Safety Translational Research Centre, University of Manchester; † Division of Pharmacy and Optometry, School of Health Sciences, Faculty of Biology, Medicine and Health, University of Manchester, Manchester Academic Health Sciences Centre (MAHSC), Manchester; ‡ Division of Primary Care, School of Medicine, University of Nottingham, The Medical School, Queen's Medical Centre, Nottingham, United Kingdom.

**Keywords:** medication safety, contributory factors, patients' views

## Abstract

Medication safety incidents are common in primary care and contributory factors frameworks can assist in our understanding of their causes. A framework that is positioned from the perspective of patients would be advantageous in practice when seeking patient insights into medication safety. The aim of this study was to develop a patient-centered contributory factors framework for examining medication safety incidents.

**Methods:**

A purposive sample of 106 members of the public, patients, and carers were recruited to take part in focus groups (n = 18). Focus groups were audio recorded, transcribed, and analyzed using a thematic framework. A patient and public involvement group was set up to undertake multiple roles in the research process, including the development of the focus group schedule, analysis of the data, and the construction of a patient-centered framework of contributory factors (patient-centered medication safety) and implementation checklist.

**Results:**

The findings highlighted the importance of communication, supplies of medication and appliances, patient- and carer-related factors, healthcare professional factors, and computer systems and programs in the safe use of medicines. Some contributory factors were unique to primary care patients such as access to services and continuity of care. In conjunction with a patient and public involvement group, a framework of factors that patients believe contribute to medication safety incidents in primary care was developed that could be used by patients and healthcare professionals.

**Conclusions:**

The patient-centered medication safety framework and implementation checklist provides a novel tool to examine contributory factors that can lead to medication safety incidents from patients' perspective.

## Abstract 2

Supplemental digital content is available in the text.

In understanding the occurrence of medication safety incidents, a so-called system-based approach to analysis has often been espoused. Such an approach typically seeks to place an incident in the context of contributory factors, usually characterized as “active failures” (actions that immediately precede the incident) and “latent failures” (contextual factors, such as characteristics of the work system or practice within it, that give rise to active failures or degrade the safeguards intended to protect against their effects). Various models have previously been proposed to help understand the contributory factors to patient safety incidents in health care. These include Reason's accident causation model,^1^ the London protocol,^2^ Systems Engineering Initiative for Patient Safety (SEIPs),^3^ and the Yorkshire Contributory Factors Framework (YCFF).^4^ Such models can be used as a template by organizations for learning about errors within their systems. Most of these models are intended for use by healthcare professionals to identify active and latent failures relating to a broad spectrum of patient safety incidents and were developed on the basis of data collected from a healthcare professional perspective.^2,4^ The exploration of patients' views of safety has, however, been largely overlooked.^5^ Current policy highlights the need for “organizations to seek out the patient and carer voice.”^5^ This need is further strengthened by the fact that patients are able to identify factors that contribute to medication safety incidents^6^ and are willing to provide feedback on the safety of their care.^7,8^ Furthermore, the factors that patients identify are often different to those identified by health care professionals.^9,10^ As such, patients provide an important and complementary view on safety and can be an extra barrier against avoidable harm.^11^

Across the various models, the patients (as well as the healthcare professional) are often featured as a potential contributory factor. To date, there has been no attempt to systematically gather patient perspectives to build a model that depicts the range of contributory factors seen by those on the receiving end of medication safety incidents. Such a model could provide a useful addition to other contributory factors models, which mainly focus on the healthcare professional perspective in the hospital setting.

An important but relatively underresearched setting in which to investigate medication safety issues is primary care. Prescribing or monitoring errors occur for one in eight patients, involving approximately one in 20 of all prescription items issued from general practice.^12^ In 2016/2017 alone, 1 billion prescription items were dispensed in primary care in England.^13^ In the United States, nearly 5% of hospitalized patients experience an adverse drug event, and it is thought that primary care patients may experience even higher rates,^14^ and in Australia, between 8.5% and 12% of people attending general practice have experienced an adverse medication event in the previous 6 months.^15^ Such figures demonstrate the scale of the problem and point to the obvious need to tackle the underlying contributory factors to medication safety incidents in primary care.

To this end, we set out to develop a patient-centered contributory factors framework of medication safety issues that could be used alongside existing frameworks to examine the patients' perspective. A framework of contributory factors that is grounded in patient accounts of medication safety and developed in conjunction with patients and the public could facilitate patient involvement in the surveillance of medication safety processes as well as assisting in the investigation of medication safety incidents.

## AIM

This study aimed to develop a patient-centered contributory factors framework and implementation checklist for examining medication safety issues that can support incident investigation and lead to improvements in understanding medication safety in primary care from the patient and carer perspective.

## METHODS

In this study, we define **“**medication safety incidents” to refer to the event that happens and “error” to refer to a type of active failure that leads to an incident.

## PARTICIPANTS

To gain an alternative and often absent insight into the factors that contribute to medication safety in the primary care setting, we sought to capture the views and experiences of patients who represent the wide range of primary care service users.

We sought representation from patients who use medications for a variety of health conditions, including patients with long-term conditions or multimorbidity, because they are at particular risk of medication-related problems^16^ but also groups that are underrepresented in patient safety research but that might encounter problems in their use of medicines and healthcare services (e.g., deaf, visually impaired).^17,18^ Carers (anyone who cares, unpaid, for a friend or family member who due to illness, disability, a mental health problem, or an addiction cannot cope without their support^19^) were also recruited to take part in focus groups. Individual patients were selected if they had a long-term condition requiring them to use multiple medications or if they had experienced safety issues relating to their medication.

They were recruited in the following three main ways: (*a*) a contact list of people who had previously expressed an interest in patient safety research; (*b*) advertisements in social media; and (*c*) local community organizations such as elder groups and support groups for people with a specific long-term condition. Mental health service users were recruited through a local mental health trust, which approached service users on our behalf. Participants received a £20 gift voucher and travel expenses for taking part in the study.

## PROCEDURE

Ethical approval for this study was obtained from London City and East Research Ethics Committee (reference 13/LO/1531). Data were collected through focus groups, with between 3 and 11 participants. The research team liaised with various community organizations and support groups to both recruit and organize focus groups. Most of the focus groups took place in the usual meeting place of the community and support organizations. A member of the research team explained the purpose of the focus group and then took consent from each participant. Focus groups were led by one of three members of the research team (S.J.G., D.L.P., or P.J.L.), with another member of the research team, or a layperson (FM) from our study patient and public involvement (PPI) group, acting as a co-facilitator. Three focus groups were conducted for participants who were unable to communicate in spoken English: one in Urdu; one in Hindi; and one in British Sign Language (BSL). For the first two of these groups, a researcher who was fluent in the respective language acted as the lead facilitator, with a member of the research team as co-facilitator. The third group was facilitated by D.L.P. with the assistance of a BSL interpreter, who provided a contemporaneous commentary of the discussion.

A semistructured topic guide was developed by the researchers and the PPI group members to address the research question (see supplementary file 1, http://links.lww.com/JPS/A224). The following topics were included: problems that participants had experienced with medications; their interactions with doctors and pharmacists with regard to medications; their own contribution to the safe use of medications; and their knowledge of adverse event reporting.

Focus groups were audio recorded and fully transcribed. They were between 75 and 120 minutes in duration.

### Data Analysis

Focus group transcripts were imported into NVivo 10 (version 10, 2014; QSR International Pty Ltd, Sydney, Australia). A thematic approach^20^ to analysis was taken with coding of the data occurring both deductively through a thematic framework of existing contributory factor models^2–4^ and via a bottom-up, inductive approach in which new codes were derived from initial readings of the focus group transcripts. Initially, two members of the research team (S.J.G. and P.J.L.) separately reviewed the same three transcripts within the data set to identify contributory factors to medication safety incidents and to agree on the coding framework for the analysis. Subsequent transcripts were coded using the agreed framework (based on both a priori and emergent codes) and the analysis undertaken by S.J.G. and P.J.L. A member of the PPI group was also asked to use the coding framework to code the data from the initial three transcripts and to explore whether their coding was similar to that of the researchers. There was little variation in the coding undertaken by FM with that of S.J.G. and P.J.L. (the researchers). Any variation was resolved through discussion.

#### Patient Involvement

In addition to the focus groups, a PPI advisory group was set up as part of the project. This group consisted of eight members of the public who, either through their own contact with primary care services or in their capacity as a carer, had experience of taking or assisting a relative to take multiple medications. They were all paid £20 per hour for their time and given travel expenses. However, a key part of their role was to contribute to the design of the patient-centered medication safety (P-MEDS) framework and implementation checklist.

Eight members of the public were involved in the design of this study, five females (age, 25–65 years) from white British and Afro Caribbean backgrounds and three males (age, 30–55 y) from white British and Indian backgrounds. They were involved in commenting on and suggesting changes to the study protocol. They helped formulate questions for the focus groups and suggested changes when the meaning was ambiguous. One member (F.M.) was involved in the analysis of focus group transcripts, we held a training session with her to ensure that she was able to undertake this task. They were also involved in all meetings about the study, where they were able to suggest possible community groups to include in the study and help recruit them. During the data collection phase, the members of the public were involved in co-facilitating focus groups. This often helped put participants at ease, because there was also a member of the public present, which reduced the formality.

Four members of the public from the patient advisory group (1 male and 3 female) were involved in helping the research team design the P-MEDS framework by commenting on its visual appearance, the words used to describe the issues contained within it and the definitions used. These same four members also co-designed a checklist to help operationalize the P-MEDS framework, along with S.J.G. and P.J.L. This was to ensure that the P-MEDS checklist was comprehensible. The final version of the P-MEDS framework and checklist was a result of both the data from the focus groups as well as input from the PPI advisory group.

In collaboration with the PPI group, we have produced a summary of the findings, which will be sent to study participants.

## RESULTS

### Participants

Data were collected via 18 focus groups with 106 participants. They were organized around a particular demographic group or condition, including elders (Asian, Caribbean, and white), patient and parents of children with long-term conditions, transgender, patients with heart disease, renal disease or mental health problems, those recovering from drug addiction, deaf people, and those with visual impairment. Table 1 shows a breakdown of the groups that took part.

**TABLE 1:** Focus Group Participants

Participant Type		n
Patients with long-term conditions (including diabetes, epilepsy, cardiovascular disease, cancer, and overall multimorbidity) (2 groups)		18
Parents of children with long-term conditions		4
Renal patients		8
Cardiovascular patients (2 groups)		19
Mental health service users (3 groups)		11
Recovering from substance misuse		6
Male-to-female transgender		3
Deaf service users		6
Visually impaired service users		4
White elders (2 groups)		6
Caribbean elders		9
Asian elders	(Urdu)	8
	(Hindi)	4
Total		106

From analysis of the focus group data, it was clear that participants had an understanding of the issues they faced regarding their medication and were able to both identify and discuss factors that contributed to medication errors in primary care and across transitions into primary care as well as being able to identify actual medication errors.

Table 2 summarizes the issues raised by patients. Quotes from the focus groups are used to illustrate the ways in which patients referred to the contributory factors. There were three issues not covered by other contributory factors frameworks that emerged from the analysis. These were access to services, continuity of care, and computer systems and programs. The issues that were most frequently identified were communication issues, supplies of medication and appliances, patient- and carer-related factors (such as patient knowledge), healthcare professional factors, computer systems and programs, and supplies of medication and appliances. Less common contributory factors included the following: dignity and respect, roles and responsibilities, workload of the healthcare professional, medication policies and processes, medication safety culture, and continuity of care. Active failures were also mentioned and included prescribing errors and dispensing errors.

**TABLE 2:** Contributory Factors: Issues Identified

Issue	Definition	Illustrative quotes from interviews
1. Access to services	Access to services that provide medicines and prescriptions, and/or access to health care professionals who can give you information about medicines.	*“So, I can't get my full medication which involves a 12 mile round trip which… and part of the reason I take the medication is because of pain in my back, and the drive causes even more pain in the back, there's a little irony for you.” FG15 R1*
2. Communication	Effectiveness of the exchange and sharing of information about medicines between hospital and general practice, staff, patients, groups, departments, and services	
a. Communication between HCPS	Lack of effective communication in supplies of medication, changes in dose, formulation	**Hospital/GP communication** ***“**I don't know if everybody else's GP is the same, but mine now, the repeat prescriptions are very much computerized, and if the prescription says you take one tablet a day and you've had 28 tablets. The hospital says, look I need you to take two tablets a day. You put a new prescription in, not only do they not give it to you when you go to pick your prescription up, they don't even tell you they're not giving it you. You know, they don't ring up and think, hang on, you've asked for this, and according to the computer you're not due these for another two weeks. They just don't give them you.” FG4, R3* **Access to info** “*Now, there's a point, because there's… you've actually hit on something there, because maybe you go to the doctor, one day you go to the pharmacists, and then the next day you've got another problem, you go to the doctor, and then you go and pick it up at the pharmacist. Will notice here that the GPs sometimes cock up and that the pharmacists are more aware, wouldn't it be relevant for the pharmacist to know exactly what you're taking and have a record of it? Access to the GPs record, just of the drugs which are being prescribed.” FG15, R2*
b. Communication between healthcare professionals and patients	Lack of appropriate information about medication use, such as medication changes, length of treatment, lack of listening to patient's concerns about their medication (including medication errors)	**Communication—not counseling patients** *“And it said like, you know, you can't take this, you can't have… there was quite a lot of stuff I couldn't do. And it scared me a little bit. So I actually asked the pharmacist. I said, “can I take paracetamol on this?” She said, “no you shouldn't do, it can cause internal bleeding.” Nobody told me. I've been on it, I'm still on it now.” FG13 R2* **Length of treatment—not communicating** *“I was put on this tablet. It's to protect my stomach from all the other tablets I'm taking and I knew that it's not a long-term thing. You're not supposed to take it for so long. The GP didn't tell me that until I read up about it and it's just a short term thing. So I just take it when I need it but sometimes I get reflux and I take one then but when he prescribed it he didn't say anything about that.” FG12* **Lack of follow-up** *“I feel as though there's no back up with the doctor to see that you are taking… because I'm on about four or five different tablets and they never ask you to bring them in and to see if you're taking them regular. I feel as though there's a loophole there on the tablets I was taking.” FG8*
		**Lack of information about supplies of medication** *“R1: I'd tell her to get another prescription from her doctor to cover that and take that in, because then it's an extra to the routine one that the pharmacist has to...* *R2: That's information is it laid out for people is it?* *R1: No, it isn't laid out for people, but I think that is the answer.” FG8* **Lack of information regarding changes to medication** *“R: I found that if you change your medication, especially if you're going on a higher dose, the pharmacy ask to speak to you. I've been took in the room several times and he's gone through, he knows I've been put on a higher dose.* *I1: And, how have you found that, has that been helpful?* *R: Fine, absolutely fine. I think, well that it should be easier in your mind, because the pharmacist explained, your doctor doesn't explain. He doesn't tell you, well I'll give you a higher dose that will do it, he doesn't, but or didn't in my case, but the pharmacist sat me down in the room each time my dose of whatever it was, he took me in the side room and explained.” FG8* *them three times, it was in the medical notes, that I was allergic to, and it took a bit of a mutiny for me, plus a follow-up letter from [hospital], to get them to think that having played this game for 26 years I might know a bit about it…. But, it was killing the new graft literally, the creatinine levels were going silly. I couldn't breathe, I couldn't walk from here to that door, and for somebody that has been across the top of the Pyrenees on more than one occasion, it just wasn't right, but they were not listening.” FG4, R3* *“So they [the doctors] keep on prescribing that and you're endlessly telling them that no, I'm not taking these any more….”FG4, R6* **Dispensing error – dosage error** “Then again another time I went and I got some tablets off them, one three times a day. I said no, I'm sorry, it's one, once a day. ‘I know what I'm doing, its three times a day.’ So, I went home and got my old packet and took it in and said, what is that then? Oh, one a day, oh you're probably quite right. So, that was two occasions of pharmacies I've been and the first one was very serious.” FG
3. Computer systems and programs	Failures of systems, poor design, and lack of interfacing between systems	*“It seems to me that they've introduced a new computer system at our surgery and what it involves is it's basically a business concept called Just in Time. Basically you get your prescription just when you need it, and if there's any kind of problem whatsoever from a transport strike or anything like that you're going to miss your medication. They have difficulty adjusting even to Easter holidays. I think it was either my son or my wife, they had a prescription due on the Monday which was the bank holiday and they couldn't get the prescription because it wasn't due then. So it's become quite a problem. The chemist is absolutely sick to death of it.” FG1 R1*
4. Continuity of care	Continuity of health care professionals who deal with medicines (e.g., locum pharmacists and GPs)	*“The chemist that I use is not owned by one person. There's quite a few people coming in and out. So sometimes I get all the prescriptions, even though I've not ordered them, all at once, and I still get this amlodipine, which I don't need but I still get it. So it's just crazy. And then other times, like at Christmas, you don't get any medication at all. They've lost it.” FG1*
5. Dignity and respect	Associated with feeling comfortable, in control and valued	*“I use the usual pharmacist but I went to a pharmacist I've never used before and I handed the prescription over and she looked at me and tutted and then went over to the pharmacist and the pharmacist came back to me and said, oh, what are these? And I said, they're my tablets, they're normal tablets. Oh, well we don't stock these for these sort of people.” FG10, R1*
6. Healthcare professional factors		
This tended to focus on the knowledge of HCPs from the patient's point of view, but also about attitudes.	Characteristics and knowledge of the person delivering care that may contribute in some way to active failures regarding medicines, e.g., inexperience, stress, personality, attitudes	**Knowledge of HCPs** *“I think a lot more pharmacists tend to know more about tablets than doctors themselves, because the doctor prescribing two different tablets for two different things, they shouldn't have said well take them and take them. They should know if there's any side effects from the two tablets, which obviously they never thought of. Just well, that will do for that and that will do for that. But, you would have thought they would know you don't mix the two together.” FG15, R4* *“I like it though if the doctor puts you on something new, sometimes they don't always check whether it will conflict with something that you're already on. So, when I go to the chemist he will say to me, are you still taking… So, I think the pharmacy is really good in that respect, it's not always checked at the doctors that you could have a reaction or something.” FG8* *“I: So how does it feel when you go to the GP and you're not getting the advice that you think you need? What's that like?* *R: Frustrating.* *R: What do you mean, about the medication?* *I: Yeah.* *R: They're not really telling you, they're just prescribing.* *R1: To be truthful the doctor doesn't know as much about the tablets as the chemist, as the pharmacist because that's what they study, you know. So if you go in with a prescription from the doctor to the pharmacist they will be more able to explain more to you about it.” FG12* **Sensitivity (attitudes)** *“… I was asked, not by the pharmacist, but by the shop assistant if you like, why are you taking these? And I can only say I complained strongly at that point. I had a quiet word in a private room with the pharmacist. He eventually apologized. Did actually say that we, as a practice, have never had a trans person in before. I didn't really think that was a good enough excuse, if I was honest. I told him so and from that date onwards, up till six months ago, I've always gone back there because I used to receive a really, really good service. Now whether or not that is because I put my foot down, if you like, in the first instance, but I know a lot of trans people tend not to sort of complain, if you like. They'll just go with the flow and do that little bit of suffrage because of that. I think it's wrong that the chemist, if you like, that you go to… I understand that they need to be aware of what medication you're on, but they've really got to do it in the right way.” FG10, R2*
		
7. Medication policies and processes	Policies/directives that impact on the safety of medication usage	**Policies relating to supply of medication** *“I'm suffering from mental health problems, and my wife's got severe mental health problems, and it's very stressful, and every 28 days we have to start panicking about it. I have to have a list, I'm given a little list by Ann of exactly what medication we should be on repeat prescription. I have to check them off from the doctor and if anything's missing I have to have a word with the receptionist, and they might say oh no, you're not due that until tomorrow, so therefore you can't pick it up from the chemist until tomorrow. That's happened quite a lot.” FG1 R1*
		**Competing priorities** *“I think the big dilemma is the pharmacist is a professional sort of person and they can stand in their bit and they control all their medicines, but then the bigger the organization they work for the more sales staff they have, and that's where the weakness lies. If that person's more concerned about selling you shampoo than dispensing your drugs they're not likely to get it right. If the pharmacist hasn't got it right in the first place or if the pharmacist has made a mistake or they can't find it.… When the shop becomes a big shop and the shop becomes a big chain it gets even worse because the arm of management gets further and further away.” FG1 R8* **Resources** *“And I say, the resources aren't there anymore, where there was a time that the district nurse would come around and just change that [check what ‘that’ is].” FG12 R5* **Delay in test results due to nonalignment of systems** *“No, because like with it being a bank holiday at the time, it was a very busy clinic. Like another thing that annoyed me with them is that they take your bloods but they're not giving you the results, it's for your medication basically, so that if your potassium or your creatinine is higher, even like that, they can't sort your tablets out till the next time you go to clinic and by that time then you could be fine. FG4, R8*
8. Medication safety culture	Organizational values, beliefs, and practices surrounding the management of medication safety and learning from error	**Learning from mistakes** *“I think we should highlight the concerns either to the pharmacist or the pharmacist to the GP, like in our experience, but in terms of if we were going to kind of report it, in terms of, if it's happened once and there's a chance it could happen again, because you know that it's just a failing thing, then yeah, there should be an easier way, an easier route to report back. That's, and I don't mean that in a, to kind of have a go at GPs or have a go at pharmacists, but if it's happening on a regular basis, especially in certain GP practices, then in a way that really should be kind of looked into. Because they are so overstretched. We know that. And we're human at the end of the day. Mistakes are going to be made. But if the same mistakes kind of keep happening then that's a risk, that's a high risk and it's a, lots more patients.” FG13 R4*
9. Patient- and carer-related factors	Those features of the patient that make caring for them more difficult and therefore more prone to error. For example, abnormal physiology, language difficulties, personality.	
a. Patient knowledge		**Lack of information to use knowledge—links with poor communication of information from HCPs to patients** *“And you want to know if that's all right. And it said like, you know, you can't take this, you can't have… there was quite a lot of stuff I couldn't do. And it scared me a little bit. So I actually asked the pharmacist. I said, can I take paracetamol on this? She said, no you shouldn't do, it can cause internal bleeding. Nobody told me. I've been on it, I'm still on it now.” FG13 R2* **Knowledge not acted on** *“And this I think is the risk of real complexity in a drug regime that people don't understand the individual items they have to take, and they do their own variation of taking one of everything in the morning.” FG1 R4*
b. Patient responsibility		*“It was always my initiative of reporting side effects and it was, since then they put me on, after that they put me on another drug which worked, but in fact what I've done since then is I've stopped taking them at all because my blood pressure's gone down. And part of that is losing a bit of weight and part of it is getting rid of some of the stress that was, that I had. But it's that sort of, as a patient I realize I've got to take the initiative [laughter]….” FG10, R3* **Barriers to taking responsibility** *“With this new system that we've got it's almost impossible to manage your own condition. For instance, you've got pain killers and you're managing. Say some days you're feeling better and other days you're not. You put your prescription in, and when they started up you couldn't get a new prescription because you'd used too many of the other tablets and not enough of the other ones, and they said right, fair enough, you're not getting it.” FG1 R6* **Lack of responsibility** “Well, personally I never found it difficult because I think my doctor just tells me why I'm having it and you accept it, don't you, once you've been told.” FG8
c. Patient involvement		*“Just produced by different manufacturers and sold under different brand names, which is a curiosity I feel. And anyway, things went on with this and anyway I ended up just dropping this particular drug because I didn't think it was going any good. And well the whole, the side effect went away [laughter].” FG10, R3* **Lack of acknowledgment of adverse effects** *“The ultimate decision I found is with yourself. The medical profession don't want you to come off the drugs or don't want to change the drugs willy-nilly. And when you find something is reacting with you or not performing as you would've hoped, getting it changed or coming off it is a very, very difficult thing to progress for the reasons that us have all said, because you end up essentially making the decision yourself that that drug is bad for me, I'll come off it. And then you're put under immense pressure to go onto an alternative or another form of drug.” FG9* **Patient decision to stop taking medication because adverse effects** *“I went to the doctor. She changed my blood pressure tablet and the tablet that she gave me I took one. When I… I took this tablet in the morning and when I went out in the street I was going like this and staggering and I could hardly walk…. So I didn't take any more. I went back to the doctor. She said, what's wrong? I said, well it makes me dizzy and I'm staggering, I have no energy or anything. She said, it shouldn't be. I said, well I'm not taking any more, no way am I taking it. So I gave it to her and she changed it to something else, you see, and from when she changed that I'm alright, you see….” FG12*
d. Physical and cognitive	Patient condition affects safety or impact of safety	*“It's almost one of my hobbyhorses. I have a big issue around if you are blind or partially sighted how do you read your medication label if you don't read braille? Because a lot of people assume that the braille on packaging is the detail, but it isn't, it's the product. So when the pharmacist puts a sticky label on the packet for the individual, if you can't see that label, which is virtually anybody who is sight impaired, then actually how are they going to be compliant. So I can't read those medication labels. It's a particular issue even when I had better vision, in that it's usually printed in economy print.” FG19*
10. Role and responsibilities	Existence of clear lines of responsibility clarifying accountability of staff members and delineating the job role when dealing with medicines (complaints and lack of clarity around lines of responsibility)	*“… if you have somebody who might be elderly, okay, and can't manage their medication themselves, it's always kind of that hot potato as to whose responsibility it is in order to help facilitate that administration of that medication. So is it health's responsibility for a district nurse's prompting, or does it go back to social care? And it goes back to social care each and every time, and them that is what's landed is that the person who is the customer then has to pay for somebody to come and administer their medication that's been prescribed. They have to pay for that service weekly. When really, is that really something that is a social need, or is it a need for the health service? That's kind of my only bit of beef really, where medications and safety is concerned. Otherwise, and if people don't want to pay for that service then, you know, what happens? They don't get that service. They'll stop them coming through the door and then there's a risk.” FG13 R4*
11. Supplies of medication and appliances	Issues surrounding obtaining timely supplies of medicines or appliances	*“… so I end up having to deal with it with colchicine, providing the gouts staying away, you take one tablet a day. If you get a couple of bad doses you'll be half a dozen every day for a couple of days to get rid of it, and of course they come in batches of 100, and sometimes you've got three bottles there and you haven't touched them. I mean, colchicine is dangerous stuff, you don't want that lying around.” FG4, R3* *“I've had personal experience where a very simple medication was given me, when I went to a different chemist in a different area to actually collect the script; I was given a different medication which had an adverse effect. Sorry, the same medication but it had an adverse effect on me because of how it was put together, whatever was binding together or whatever.” FG9*
12. Workload of health care professionals	Perceived level of activity and pressures on time during working and hours	*“Our pharmacy was really stressed at Christmas, you could tell. There were massive queues, you know, because everyone was trying to get their prescriptions in before Christmas. And I think I had one for something, I can't remember what it was now. It might have been for Isobel, and the first thing when I handed it in, she said, please don't tell me you need this today. That's what she said. So I was like, eh, no I can wait until tomorrow, and she went, on thank goodness. She was just so stressed and busy.” FG13*

A more detailed description of the most common contributory factor domains is provided hereinafter (Table 3).

**TABLE 3:** Most Common Contributory Factors

1. Communication
a. Communication between healthcare professionals
b. Communication between healthcare professionals and patients
2. Patient- and carer-related factors
3. Healthcare professional factors
4. Computer systems and programs
5. Supplies of medication and appliances

To provide the context and in which the contributory factor domains were developed and to demonstrate the robustness of the data used to develop the P-MEDS framework, the next section will elaborate on these key themes before describing the development of the P-MEDS framework itself.

### Communication Issues

Communication problems were the most commonly cited issue by focus group participants. These issues related to two main types of communication: communication between healthcare professionals (including pharmacists and doctors) and communication between health care professionals (HCPs) and patients.

Participants commonly described the poor communication between health professionals and how this could impact on their safety. Communication problems were reported between general practitioners (GPs) and pharmacists and also between prescribers in primary and secondary care:

“But the GP won't make a decision on whether I should still be on them because I'm still under the hospital, and the hospital says what are you doing on them? You should've been off them.” (Heart Group)

Communication between healthcare professionals and patients was often described as being problematic, for example, participants expressed concern over the lack of information about starting and stopping medications:

“I was put on this tablet…. You're not supposed to take it for so long. The GP didn't tell me that until I read up about it and it's just a short term thing… but when he prescribed it he didn't say anything about that.” (Elderly Group)

In addition, participants described the poor communication with patients when dealing with errors:

“… she [the pharmacist] said “it's three times a day”, and I said “no, no, one a day.” “No” she said to me, “I know what I'm doing.” I thought well, I don't think so. I didn't argue I just went home and got the box and presented it….” (Heart Group)

Patients described occasions in which healthcare professionals did not listen to them and how this could result in mistakes being made with their medications:

“The medication issues for me are mainly about doctors at the transplant unit not listening to the patient or the medical colleagues from the [hospital] unit, and deliberately giving me medication straight after the transplant. Which I had told them three times, it was in the medical notes, that I was allergic to, and it took a bit of a mutiny for me, plus a follow-up letter from [GP], to get them to think that having played this game for 26 years I might know a bit about it…. But, it was killing the new graft literally….” (Renal Group)

### Patient- and Carer-Related Factors

Patient and carer factors could be broken down into the following four subcategories: patient knowledge, patient responsibility, physical and cognitive, and patient involvement.

Although many participants described generally having a good knowledge of their medications, sometimes, they expressed a lack of information to enable them to use knowledge:

“… it [patient information sheet] said like, you know, you can't take this, you can't have… there was quite a lot of stuff I couldn't do. And it scared me a little bit. So I actually asked the pharmacist. I said, can I take paracetamol on this? She said, no you shouldn't do.... Nobody told me. I've been on it, I'm still on it now.” (Parents Group)

Another issue that was common among focus group participants was barriers to taking responsibility, such as the patient below who expresses the barriers they face with a new system:

“With this new system that we've got it's almost impossible to manage your own condition. For instance, you've got pain killers and you're managing. Say some days you're feeling better and other days you're not. You put your prescription in [but] because you'd used too many of the other tablets and not enough of the other ones, and they said right, fair enough, you're not getting it.” (Patients with long-term conditions)

Particular groups of patients had characteristics that meant that they were more vulnerable to error (e.g., deaf patients, visually impaired patients, autistic patients, elderly, and non-English speakers). For example, a deaf patient explains how being deaf can make them particularly susceptible:

“… but there's nobody to phone, sometimes I have to go and knock on next door and ask them to phone for me and that can be at any time, which is difficult… I think there is a text system, but there isn't a text number for the chemist or the doctor, you just get it for the emergency services, like, 999, but there's nothing.” (Deaf Group)

Visually impaired patients were particularly vulnerable to error and used ways to work around the difficulties they experienced. A visually impaired patient describes how they were able to recognize a mistake with their medication using a device:

“… the size of a radio, but a lever that lifts up, takes the picture and then reads what's on the box. It says Miss Philips, I thought that's not mine. So, I went to the chemist and I said, you know, your driver has just given me these and I don't think I'm a Miss as far as I'm aware.” (Visually Impaired Group)

### Healthcare Professional Factors

Another main theme from the focus groups was healthcare professional factors, which are characteristics and knowledge of the person delivering care that may contribute in some way to medication safety incidents. These issues tended to focus around two main themes: attitudes toward patients and expertise/knowledge of healthcare professionals.

One participant from the transgender group describes an issue that they had experienced relating to the attitude of a pharmacist:

“… I was asked, not by the pharmacist, but by the shop assistant if you like, why are you taking these? And I can only say I complained strongly at that point. I had a quiet word in a private room with the pharmacist. He eventually apologized. Did actually say that we, as a practice, have never had a trans person in before. I didn't really think that was a good enough excuse… I understand that they need to be aware of what medication you're on, but they've really got to do it in the right way.” (Transgender Group)

Other participants expressed concern in relation to the expertise of healthcare professionals, particularly in relation to interactions between drugs and their lack of knowledge on this topic, as one participant describes below:

“… if the doctor puts you on something new, sometimes they don't always check whether it will conflict with something that you're already on. So, when I go to the chemist he will say to me, “Are you still taking….?” So, I think the pharmacy is really good in that respect, it's not always checked at the doctors that you could have a reaction or something.” (Heart Group)

### Computer Systems and Programs

Electronic systems used within the primary care setting were cited as a factor in medication problems. Such systems were either poorly designed, leading to problems with medication supply or were not kept updated with changes to patients' medications:

“… they've [GP surgery] introduced a new computer system at our surgery…. Basically you get your prescription just when you need it, and if there's any kind of problem whatsoever from a transport strike or anything like that you're going to miss your medication. They have difficulty adjusting even to Easter holidays…. So it's become quite a problem..” (Patients with long-term conditions)

### Supplies of Medication and Appliances

Participants often expressed difficulties in accessing medications in a timely manner, which could lead to patients missing their medication:

“I've not had my GTN spray. They've [pharmacy] not ordered it for some reason, even though I've ticked the box. It used to be that you could have them two weeks in advance, you could have your prescription and you'd have two weeks in hand. Now I'm lucky if I've got an hour in hand.” (Patients with long-term conditions)

The nature of the supply system for medications means that patients often receive medications made by different manufacturers. Previous studies have discussed how generic substitution can cause confusion for patients^21^ and can be a factor in medication errors.^22^ In one example, an error was not spotted by the mother of a patient because she was so used to receiving medications that appeared different:

“And they were always changing the packaging… the little brown things that the pharmacy send, it was called Bio-Melatonin and it was a different name and they were always, they were quite often changing the milligrams so I suppose I didn't even, and I didn't know they did a slow release one.” (Parents Group)

### How P-MEDS Was Developed

**FIGURE 1:**
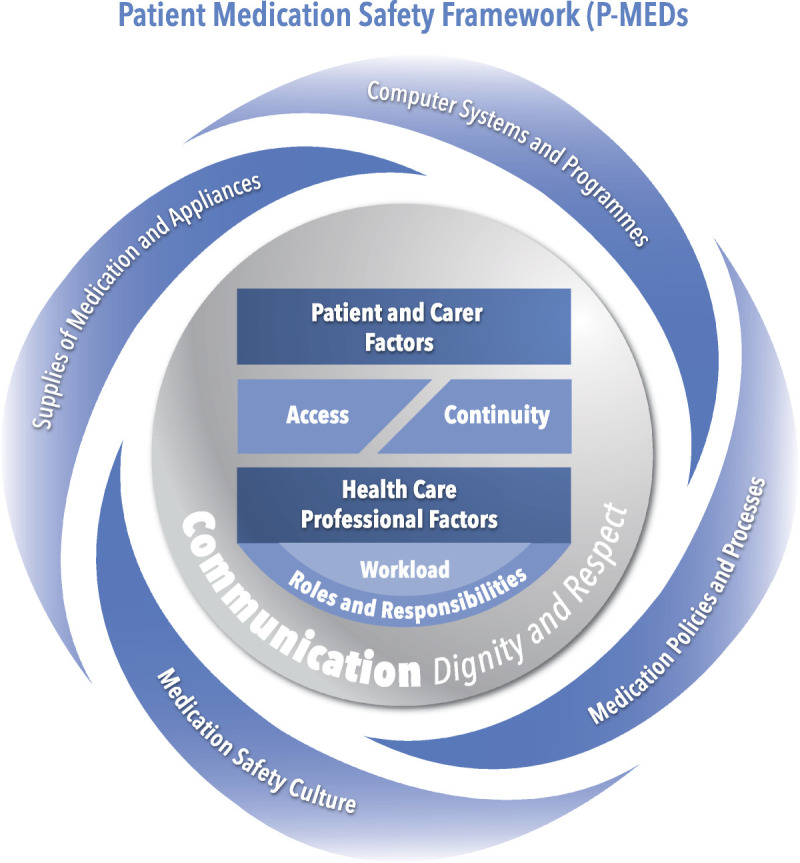
Patient medication safety framework.

**FIGURE 2:**
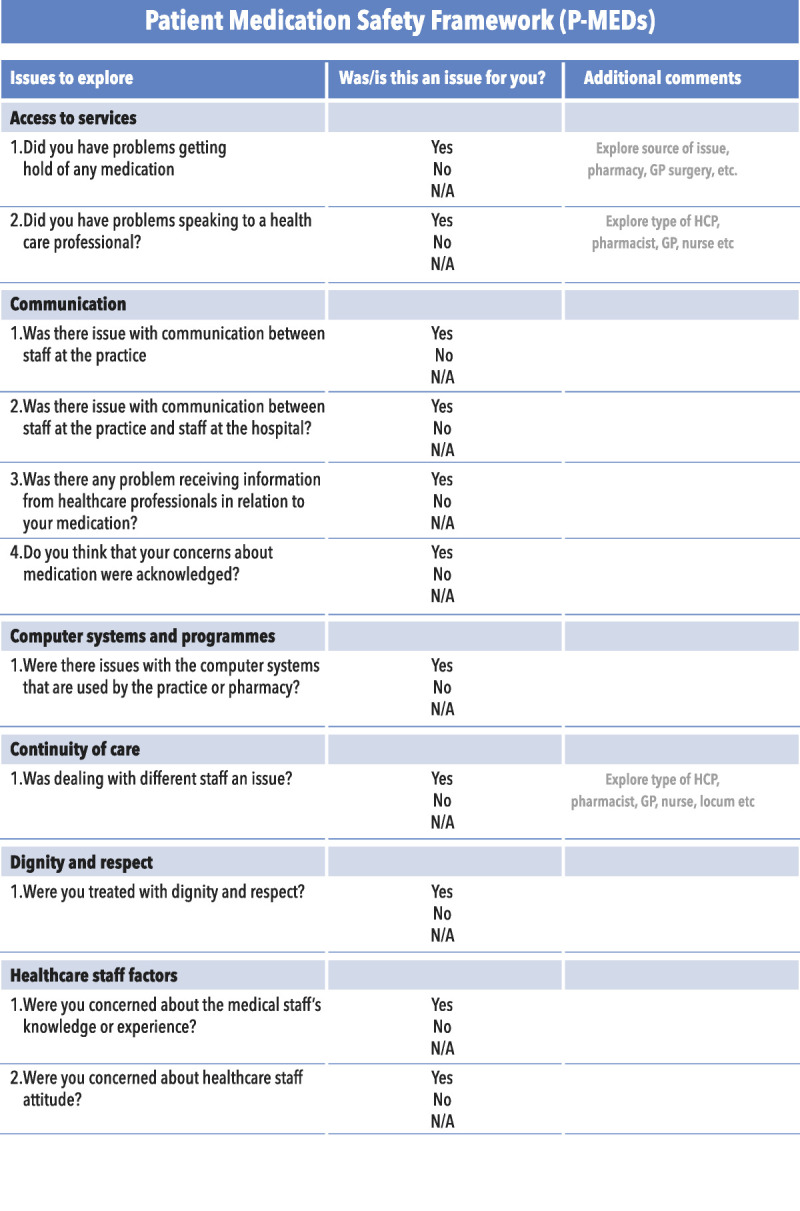
Checklist for P-MEDs.

In collaboration with eight members of the public, a P-MEDS and checklist was produced (Figs. 1, 2). This process involved five face-to-face meetings between the research team and members of the public who were presented with different versions of the framework along with the definitions of the contributory factors until agreement was reached on the final version of P-MEDS. Patient-Centered Safety Framework brought together the contributory factors raised by patients (Table 3), and by working with patients in its development, the framework and checklist were developed to ensure that they were comprehensible by patients and the public. In the P-MEDS model, communication is depicted centrally, because it was the most common theme to emerge from the focus groups with all other issues inextricably linked to this concept. A list of definitions for each of the factors within the framework is also provided (Table 4).

**Figure:**
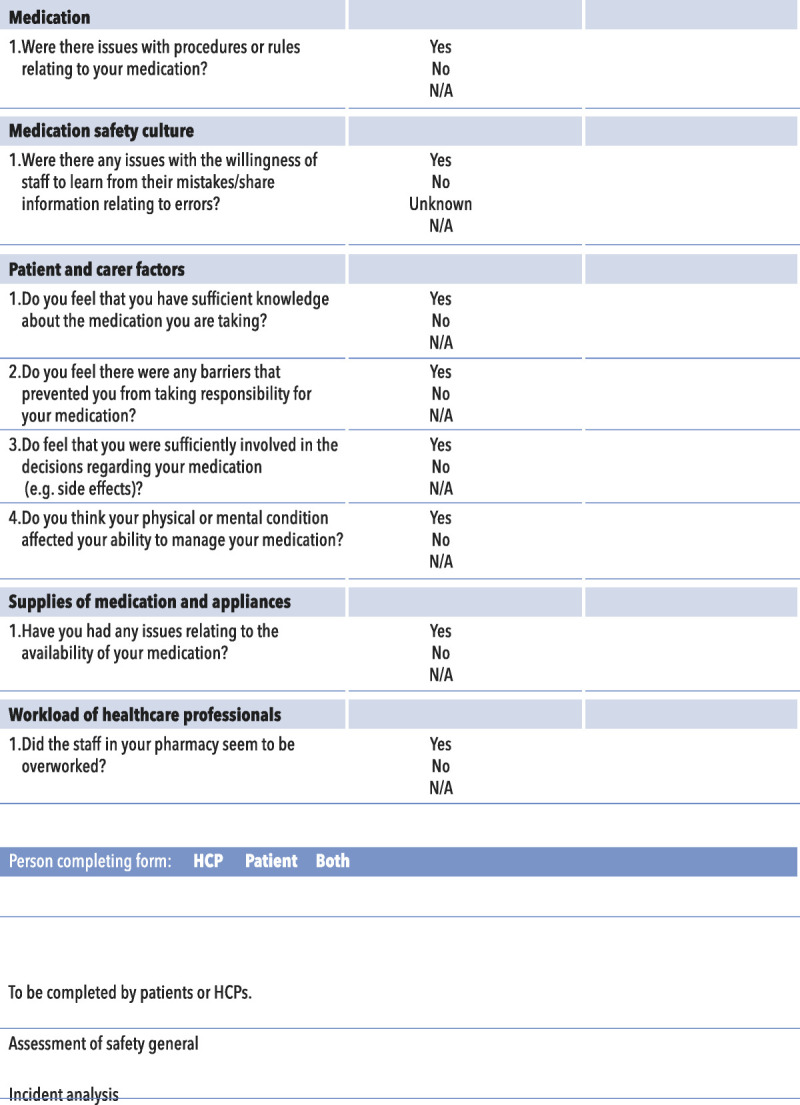

**TABLE 4:** Patient-Centered Medication Safety Definitions

Issue	Definition
1. Access to services	Access to services that provide prescriptions and medicines, and/or access to health care professionals who can give you information about medicines
2. Communication	Effectiveness of the exchange and sharing of information about medicines between hospital and general practice, staff, patients, groups, departments, and services
a. Communication between HCPS	Lack of effective communication in supplies of medication, changes in dose, formulation
b. Communication between healthcare professionals and patients	Lack of appropriate information about medication use, such as medication changes, length of treatment, lack of listening to patient's concerns about their medication (including medication errors)
3. Computer systems and programs	Failures of systems, poor design, and lack of interfacing between systems
4. Continuity of care	Continuity of health care professionals who deal with medicines (e.g., locum pharmacists and GPs)
5. Dignity and respect	Associated with feeling comfortable, in control and valued
6. Healthcare professional factors	Characteristics and knowledge of the person delivering care that may contribute in some way to issues with medicines, e.g., inexperience, stress, personality, attitudes
7. Medication policies and processes	Policies/directives that impact on the safety of medication usage
8. Medication safety culture	Organizational values, beliefs, and practices surrounding the management of medication safety and learning from error
9. Patient- and carer-related factors	Features of the patient that makes involvement in safe use of medicines more difficult and therefore more prone to error (e.g., abnormal physiology, language difficulties, personality)
a. Patient knowledge	
b. Patient responsibility	
c. Patient involvement	
d. Physical and cognitive	Patient condition affects safety or impact of safety issues
10. Role and responsibilities	Existence of clear lines of responsibility clarifying accountability of staff members and delineating the job role when dealing with medicines (complaints and lack of clarity around lines of responsibility)
11. Supplies of medication and appliances	Issues surrounding obtaining timely supplies of medicines or appliances
12. Workload of health care professionals	Perceived level of activity and pressures on time during working and hours

## DISCUSSION

Patients' experiences and views on medication safety in primary care provide an important insight into safety that can be used by primary care practitioners in their efforts to reduce medication errors in this setting. However, current models that support practitioners with unpacking those factors that can lead patient safety incidents are not designed with this purpose in mind. Therefore, we set out to develop a patient-centered framework and associated checklist that can be used in practice to capture the patient perspective on aspects of medication safety. The tool, comprehensible by practitioners and patients alike, will help facilitate patient involvement and complement existing tools to provide a more complete understanding of medication safety issues within the primary care setting. The foundations of P-MEDS rest on a diverse group of patient perspectives that together provide a more holistic view of contributory factors to medication safety incidents that was further strengthened by including patients in the development of the P-MEDS framework and checklist.

Our findings highlighted some key themes related to patient-centered medication safety, and although it is not possible to discuss all of themes within the remit of this article, it would be useful to reflect on those major themes that we have presented.

It is well known that communication problems are a major issue in patient safety incidents^23^ and are one of the most frequently cited causes for patient safety incidents^24^ from the healthcare professional's perspective. As a result, there have been various tools developed to help improve communication among healthcare professionals, mainly in secondary care. These include safety briefings and clinical handover tools.^25,26^ It is therefore perhaps not surprising that communication issues were discussed frequently by all groups of patients and identified as a major issue in medication safety incidents. Patients' accounts were centered around communication failures between healthcare professionals in primary and secondary care, where they sometimes failed to share information regarding the medications patients were taking and also around the failure of healthcare professionals to share all relevant information regarding medication with their patients. For this reason, communication is depicted centrally in the P-MEDs framework. By placing communication centrally in the framework, we hope to draw the attention of healthcare professionals to the importance of communication and its role in medication safety incidents.

It is well known that patients from marginalized groups are more at risk of experiencing patient safety incidents.^27–29^ This is reflected in the P-MEDs framework, which demonstrates the importance of patient-related factors in relation to medication safety incidents. In addition, in our study, patients have recognized that both the medical conditions and knowledge they have about their condition are important factors in contributing to medication safety incidents. It is for this reason that patient-related factors were one of the more commonly cited issues during the focus groups. Moreover, patients also recognized that healthcare professional factors relating to their knowledge and attitudes toward patients play an important part in medication-related safety incidents. By including both these issues as key elements of the P-MEDs framework, it is possible to demonstrate to healthcare professionals that these factors play an important part in contributing to medication safety incidents.

Computer systems and supplies of medications were closely interlinked. There has been discussion of how the design of computer systems can impact on safety.^30^ Patients were sometimes prevented from managing their own medicines or even accessing their medicines because of the poor design and implementation of IT systems. Supplies were also affected by pharmacy and GP procedures that could prevent timely access and dispensing of medicines.

### How Does It Compare With Other Frameworks?

In contrast to P-MEDS, most current models or frameworks^2–4^ are created by or with healthcare professionals and have minimal patient input. We sought to elucidate the patient's perspective by focusing on the voices of more than 100 primary care patients and also through the guidance of Patient and Public Involvement throughout the process of the study. This led to the development of not only a model in which patients feature in the constructs but also a model that is founded in patient perspectives. Because of this approach, P-MEDS captures the contributory factors that patients consider to be important to medication safety in primary care. There is clearly overlap with existing contributory factor frameworks but also notable differences such as the addition of contributory factors including “access to services” and “continuity of care.” These additional factors were important issues for patients in relation to the safe use of medicine.

Access to services^31^ does not feature as a contributory factor in other models, but it is included in the UK's National Reporting and Learning System^32^ (a central database of patient safety incident reports), which asks reporters to categorize patient safety incidents as to whether they were associated with failures with access or admission. Our study found that medication safety incidents could arise from lack of access to services and that patients believed this to be an important component of safety. In the YCFF, scheduling and bed management is a listed contributory factor,^4^ describing a similar yet hospital-based issue. Access is a factor that is related to other latent conditions within the environment such as care processes in Systems Engineering Initiative for Patient Safety,^3^ organizational and management failures, and institutional context factors in the London protocol^2^; however, the problem, as perceived by patients and that is proximal to the medication incident, is “access.” Patients can identify with this term but clinicians when using P-MEDS in conjunction with other tools or models would likely detect those more distal factors cited in traditional frameworks.

Continuity of care is known to be important to patient satisfaction^33^ and has also been shown to impact on mortality.^34^ There are related issues cited in other models such as “shift patterns,”^2^ “organizational structure,”^2,3^ and “team structure,”^2^ but as with “access,” these are factors that could be identified by healthcare professionals that work within the system but difficult for patients to identify. What is important to patients is continuity, which can emerge as a result of some of these latent factors within the care system. A model such as P-MEDS allows patients to spot the symptoms of these latent factors, which otherwise may go undetected.

Other differences between P-MEDs and other models relate to the types of contributory factors that patients are able to identify, for instance, the London Protocol^2^ includes “work environment factors” and Systems Engineering Initiative for Patient Safety the component “organization.”^35^ Both of these terms relate to a broader set of issues including workload, supervision, skill mix, and management support, which are difficult for patients to recognize and were therefore absent in focus group discussions. Patient-centered medication safety focuses on the one aspect that patients did observe and discuss in relation to medication safety and that was the issue of workload. It was possible through patients' direct observations or discussions with healthcare professionals that workload featured as a factor in their descriptions of medication safety incidents. As with P-MEDS, the YCFF^4^ includes staff workload in its model but redundant from our model were other elements of the YCFF, such as team factors, and supervision and leadership. Potentially, patients in the hospital setting may detect such factors because they are constant observers within the ward. However, our study focused on the experiences of patients in primary care, although their experiences were influenced by previous episodes of hospital stay and the difficulties in the transition from hospital care to general practice, and they focused on their observations and experiences of medication safety in primary care. Furthermore, the YCFF is a hospital-based tool developed based chiefly on research studies with health care professionals' incident reports, which also meant that it included factors such as scheduling and bed management and support from central functions—all of which inhibit their usefulness in the primary care setting. There is also the Linneaus Framework,^36^ which like P-MEDs is a primary care framework but Linneaus focuses on classifying patient safety incidents rather than exploring the contributory factors that might give rise to an incident so it has limited usefulness in understanding the array of issues that give rise to errors. Importantly, all of these current models are generic—they can be applied to any type of patient safety incident, but in doing so, they lack the specificity and terminology that might be useful in helping patients and healthcare providers identify what went wrong in the medicines use process.

### How Could P-MEDs Be Used?

Despite the current drive toward involving patients in the investigation of error,^10^ there is a need for more structured guidance on how to facilitate this involvement. Patient-centered medication safety and its checklist could be used by practitioners to bring the patient more centrally into the medication safety incident investigation process. The checklist could be used as a set of prompts completed by patients themselves while reporting incident or working through in conjunction with an investigation team as part of an investigation. By using the checklist, it would be possible for patients to identify the array of contributory factors mitigating the potential to focus on the most immediately obvious cause. The findings of the checklist could then feed into a wider analysis of safety incidents, which may use complementary models of contributory factors. Patient-centered medication safety could also be used as a patient-orientated tool for the proactive surveillance of latent and active failures or to facilitate patient input into the design of safety systems. In such applications, P-MEDS would help patients articulate their views about the safety level in a given healthcare organization or setting, by providing them with a vocabulary and a set of concepts with which to identify contributory factors.

### Strengths and Limitations

The main strengths of our study are that it draws upon the patient's view of medication safety in a primary care setting. Patient-centered medication safety is the first patient-focused contributory factors framework and a wide range of views were captured to assist in its development. A limitation of P-MEDs is that it *only* captures the views of patients and not health care professionals. However, we do not suggest that this model is used in isolation and that other models can be used in conjunction with P-MEDS to seek out the views of healthcare professionals and other stakeholders. What is known is that the patient's voice can be absent from patient safety incident investigations, in the design of safety systems and in safety surveillance, and the use of our checklist could strengthen such processes if used alongside other tools and frameworks.

Another limitation of the study is that it only captures patients' views on medication safety incidents in primary care and therefore may not be transferable to other types of patient safety incidents or settings. However, some of the key issues it raises particularly around communication cross the interface of care contexts. We also plan to test the framework in practice, part of which will allow us to determine its transferability to other types of patient safety incidents.

## CONCLUSIONS

Patients are a valuable source of information about medication errors and the factors that contribute to them in primary care. However, to date, there is no framework of contributory factors available to assist in elucidating patients' perspectives on this important topic. The P-MEDS framework and checklist provide a lens through which patient identified contributory factors can be captured. Patient-centered medication safety can be used alongside existing approaches to ensure that patient perspectives are effectively integrated into the holistic analysis and subsequent prevention of patient safety incidents.

## Supplementary Material

SUPPLEMENTARY MATERIAL
