# Supplementary material for: Capturing Patients' Perspectives on Medication Safety: The Development of a Patient-Centered Medication Safety Framework
Source: J Patient Saf. 2019 Mar 15;16(4):e324–39. doi: 10.1097/PTS.0000000000000583 (PMC7678656; doi:10.1097/PTS.0000000000000583)
Supplement: SUPPLEMENTARY MATERIAL [file pts-16-e324-s001.docx]

**Medication safety focus group questions**

**Background**

You are likely to use services in primary care (e.g. GPs; community pharmacists; district nurses; practice nurses; care home staff). We want to know ways in which these services could be improved, especially with regard to the prescribing, dispensing and use of medicines. In other words, how to avoid things such as:

- Getting the wrong type of medicine;
- Getting the wrong dose;
- Getting someone else’s medicine by mistake;
- Being left on a medicine longer than you should;
- Medicine being changed without your knowledge.

The aim of this focus group is to get your views about medicines: what do you know about them; how easy would it be for you to identify an error or mistake in your medicine; and what role (if any) would you have in preventing errors or mistakes from happening?

**Ground rules**

- You are being digitally recorded, so speak clearly and do not speak over one another;
- Please respect each others’ right to express a view, even if it differs from yours;
- We will anonymise the transcript so that nobody can be identified by name. However, please try to avoid naming specific healthcare professionals or locations;
- Everything discussed here is confidential.

**Questions**

1. Do things go wrong with your medicines? Why (or why not)?
2. Do you think things could go wrong with your medicines?
3. What do you think about the way your medicines are handled by your GP/Pharmacist? Are there things that you like in particular, or any problems?
4. Do you have a role in helping to prevent things going wrong with your medicine?
   *Follow-up questions: How easy/difficult is it for you to do this? Are you prepared to do this? What do you do?*
5. What do you know about medication reviews? What are your views on them?
   *Follow-up questions: Do you know what they are? Do you think they are useful? How do you feel about pharmacists undertaking them? What could be done to improve them?*
6. Can service users help doctors and pharmacists to improve medication safety? How?
7. What do you think about service users reporting concerns they have about their medicines?
   *Follow-up questions: can service users report concerns about medicines? Would you be happy to report concerns that you have? Have you heard of the yellow card scheme? What would the benefits and disadvantages of service user reporting be? What would help or hinder service users in reporting concerns*
8. Are there any other issues that you think we should discuss?
